# Role of active joint position sense on the upper extremity functional performance tests in college volleyball players

**DOI:** 10.7717/peerj.13564

**Published:** 2022-06-09

**Authors:** Guillermo Mendez-Rebolledo, Amanda L. Ager, Diana Ledezma, Julieta Montanez, Juan Guerrero-Henriquez, Carlos Cruz-Montecinos

**Affiliations:** 1 Escuela de Kinesiología, Faculta de Salud, Universidad Santo Tomás, Chile; 2 Magister en Ciencias de la Actividad Física y Deporte Aplicadas al Entrenamiento, Rehabilitación y Reintegro Deportivo, Facultad de Salud, Universidad Santo Tomás, Chile; 3 Department of Rehabilitation Sciences and Physiotherapy, Faculty of Medicine and Health Sciences, Ghent University, Ghent, Belgium; 4 Centre for Interdisciplinary Research in Rehabilitation and Social Integration, Rehabilitation Institute (CIRRIS), Québec City, Québec, Canada; 5 Rehabilitation and Human Movement Sciences Department, Faculty of Health Sciences, University of Antofagasta, Antofagasta, Chile; 6 Laboratory of Clinical Biomechanics, Department of Physical Therapy, Faculty of Medicine, University of Chile, Santiago, Chile

**Keywords:** Shoulder, Proprioception, Functional performance tests, CKCUEST, YBT, SMBT, Kinesthesia, Repositioning test

## Abstract

**Background:**

It has been well established that proprioception plays a decisive role in shoulder stability and sport performance. Notwithstanding, there is a lack of clear association between active joint position sense (AJPS) and the performance of upper-extremity functional performance tests. The aim of this study was to determine whether the AJPS of the shoulder complex is associated with the performance of college volleyball players with the following functional tests: Y-Balance Test-Upper Quarter (YBT-UQ), Closed Kinetic Chain Upper-Extremity Stability Test (CKCUEST), and Seated Medicine Ball Throw (SMBT). The secondary aim was to investigate whether the magnitude of the proprioception error through the AJPS had the ability to act as a predictor for functional test scores.

**Methods:**

Cross-sectional study with a convenience sampling. Healthy college volleyball players (≥12 h of training/week), 30 males and 22 females, between 18 and 26 years of age were included. AJPS of the shoulder (90° of flexion (90°Flex), 90° of internal rotation at 90° of abduction (90°IR/ABD), 90° of external rotation at 90° of abduction (90°ER/ABD)) and three upper-extremity functional performance tests (YBT-UQ, CKCUEST and SMBT) were assessed. A Pearson’s test and a stepwise multiple linear regression analysis were used to determine possible associations and relationships between outcome measures, respectively.

**Results:**

The analysis revealed that AJPS at 90°IR/ABD and 90°ER/ABD were the only proprioceptive variables with an association to the YBT-UQ and SMBT. Despite these relationships, only the AJPS at 90°IR/ABD was associated with the performance of the YBT-UQ in; superolateral direction (β = −0.7; 95% CI [−1.3 to 0.1]; *p* = 0.025); inferolateral direction (β = −1.5; 95% CI [−2.1 to −0.8]; *p* = 0.001); and composite score (β = −0.8; 95% CI [−1.3 to −0.3]; *p* = 0.002). From these, AJPS at 90°IR/ABD mainly explained the variability of YBT-UQ (inferolateral direction) performance (R^2^ = 0.32; %R^2^ = 0.32). Our findings allow for a possible expanded role for proprioception as a contributing factor in upper limb motor control during functional movements. Further research is required to explore and distinguish the associations between proprioception, motor control and sport performance involving the upper limbs.

## Introduction

Upper extremity functional performance tests have been widely applied in overhead sports such as volleyball, handball, basketball, tennis and swimming for the evaluation of the functional performance of athletes in open and/or closed-kinetic chains of the upper extremities (Borms, Maenhout & Cools, 2016; Borms & Cools, 2018; Cigercioglu et al., 2021; Gorman et al., 2012; Taylor et al., 2016; van den Tillaar & Marques, 2013). Popular upper extremity functional performance tests include the Y-Balance Test Upper Quarter (YBT-UQ) (Cook, 2010; Gorman et al., 2012) and the Closed Kinetic Chain Upper Extremity Stability Test (CKCUEST) (Goldbeck & Davies, 2000), theorized to measure the dynamic stability of the upper extremity in a weight-bearing position (Borms & Cools, 2018; Taylor et al., 2016). Moreover, there is also the Seated Medicine Ball Throw (SMBT) test which quantifies the force and power of unilateral and bilateral throwing involving the upper extremities in an open-kinetic chain context; which is a practical functional test for athletes engaged in overhead sports (Cigercioglu et al., 2021; Harris et al., 2011). Although the YBT-UQ and CKCUEST do not specifically mimic the activity of throwing, they are important tests to consider for overhead athletes, such as volleyball players, as they test the function of the entire upper extremity kinetic chain, which is essential for the mechanism of throwing (Borms & Cools, 2018; Mendez-Rebolledo et al., 2022). In this sense, during the execution of throwing, the shoulder complex plays a fundamental role in transferring mechanical energy from the trunk and lower extremities to the ball for expulsion (Borms, Maenhout & Cools, 2020; Skejø et al., 2019). Conversely, the SMBT may be considered a functional test for volleyball athletes as it is an open-kinetic chain motor task with some characteristics similar to volleyball specific skills, such as the spike and serve (Battaglia et al., 2014; Borms, Maenhout & Cools, 2016; Taylor et al., 2016).

The current literature suggests that the performance during an upper extremity functional test can be influenced by several factors including (i) one, or the combination of, motor capabilities, such as strength, power, flexibility, coordination, among others; and (ii) the specific motor skills involved in the test or activity, *e.g*., jump, run, throw, etc. (DiCesare et al., 2019; Fernandez-Fernandez, Ulbricht & Ferrauti, 2014; Gabbett & Georgieff, 2007; Guirelli et al., 2021; Sarvestan, Svoboda & Linduška, 2020). For instance, it has been suggested that the isometric strength of the external shoulder rotators has a moderate relationship (*r* = 0.50–0.63) with the YBT-UQ in all testing directions (Guirelli et al., 2021); as well as a strong relationship (*r* = 0.75–0.79) between isokinetic strength of the shoulder rotators with the SMBT (Borms, Maenhout & Cools, 2016). However, no relationship has yet to be observed between the strength of the shoulder rotator muscles and the CKCUEST or YBT-UQ (Borms, Maenhout & Cools, 2016; Guirelli et al., 2021; Westrick et al., 2012). Nonetheless, there may be further associations between the performances of these functional tests and other motor or sensorimotor capabilities such as proprioception. In this vein, it has been suggested that proprioception could play a relevant role in the motor performance of the upper extremities during functional movements (Ager et al., 2020, 2021; Dover & Powers, 2003; Gumina et al., 2019).

Proprioception is one of the many somatosensory senses, (*i.e*., thermoception, nociception, equilibrioception, mechanoreception) that allows us to guide our body and limbs through space in the absence of visual feedback (Ager et al., 2020, 2021). Proprioception includes the sub-modalities of joint position sense, kinesthesia (sense of movement), sense of force, and sense of change in velocity (Ager et al., 2020, 2021; Lephart et al., 1997). More precisely, joint position sense is the ability to reproduce joint angles actively or passively (Ager et al., 2021; Röijezon, Clark & Treleaven, 2015). Several researchers have chosen to evaluate active joint position sense (AJPS) as a sub-modality of proprioception, as it has been suggested to be a representative variable of shoulder proprioception in healthy (Balke et al., 2011; Brindle et al., 2006; Clark, Röijezon & Treleaven, 2015; Hung & Darling, 2012) and pathologic shoulders (Guirelli et al., 2021; Lubiatowski et al., 2019). Moreover, it is hypothesized to support strong ecological validity (Gibson, 1979), as it theoretically most resembles the active functional movements of sport.

In overhead sports, proprioception could influence the final phase of throwing as it plays a vital role in ensuring the correct hand positioning and modulating the speed and trajectory of the throw (Contemori & Biscarini, 2018). By contrast, a proprioceptive deficit could alter the recruitment of the shoulder muscles, causing an over-activation or under-activation of the rotator cuff muscles, ultimately predisposing an athlete to injury (Contemori & Biscarini, 2018; Takasaki, Lim & Soon, 2016). Such injuries as a suprascapular nerve palsy, and a subsequent infraspinatus muscle atrophy, have been associated with the volleyball athlete population (Contemori & Biscarini, 2018). Such neuromuscular injuries are known to cause a reduction in proprioceptive acuity, which could ultimately alter the reflexive neuromuscular responses which protects the joint from sudden high-stressful joint loads that occur during the spike, serve, or an opponent’s block during a volleyball match (Contemori & Biscarini, 2018).

Despite the multiple investigations that indicate that proprioception is decisive for shoulder stability and sports performance, to our knowledge there is yet to be a clear association between shoulder proprioception and the performance of the upper extremities during functional performance tests. Therefore, our aim was to determine whether the AJPS of the shoulder complex is associated with the performance of college volleyball players with the following functional tests: YBT-UQ, CKCUEST, and SMBT. If any association was to be observed, it is our secondary aim to investigate whether the magnitude of the proprioception error through the AJPS had the ability to act as a predictor for functional test scores. In this way, it is our expectation to further the understanding of the association between proprioception and the performance of the upper extremities during complex motor activities which are routinely performed during sports.

## Materials and Methods

### Design

A cross-sectional study was conducted in the Biomechanics and Motor Control Laboratory of the Universidad Santo Tomás (Talca, Chile), while respecting with the Helsinki Consensus on biomedical research in humans, following the STROBE statement to report observational studies. The Scientific Ethic Committee of the Universidad Santo Tomás (Chile) approved all procedures (ID 149-19), and informed consent was given by each participant through a signed consent form.

### Participants

Following a sample size calculation (G*Power, Version 3.0.10, Franz Faul, Universität Kiel, Kiel, Germany) based on an alpha of 0.05 (error type I), a power (error type II) of 0.95, a total sample size of 70 participants, taking into account a Pearson’s correlation coefficient of 0.45, a minimum sample size of 49 participants was required for this study. Although there are no reports on the association between absolute error of AJPS and upper extremity functional performance tests in overhead athletes, we used a conservative criteria for the correlations between variables (Bujang & Baharum, 2016) and a Pearson’s correlation coefficient of 0.45, as observed in a previous study examining the relation between AJPS and upper extremity function (Ünlüer et al., 2019). Utilizing a convenience sampling, 52 amateur volleyball players between 18 and 26 years of age from a regional collegiate level league were recruited for this study (Table 1). The recruitment of the participants was carried out through announcements and meetings with the teams of the regional league. During recruitment, the following exclusion criteria were applied: (i) history of shoulder pain, trauma, luxation, and/or rotator cuff tear; (ii) reported pain with time-loss in sports participation within the past 6 months (Borms & Cools, 2018); or (iii) deformity of the spine, root symptoms and/or neurological diseases. In addition, shoulder instability was assessed using the sulcus, anterior drawer, and posterior drawer tests. The clinical evaluation of the exclusion criteria was carried out by a sports physician (8 years of experience).

**Table 1:** Baseline characteristics of the population.

	Male	Female
Number of participants (n)	30	22
Training/week (h)	16	14
Age (years old)	20.8 ± 3.2	21.0 ± 3.5
Body mass (kg)	74.7 ± 9.8	64.1 ± 6.1
Height (m)	1.74 ± 0.08	1.60 ± 0.07
Body mass index (kg/m^2^)	24.6 ± 2.9	25.2 ± 1.6
Upper limb length (cm)	90.0 ± 4.8	82.5 ± 4.7

**Note:**

Data expressed as mean ± standard deviation.

### Procedures

All procedures were completed in a single 90-min session with a 5-min rest between tests. Athletes completed a general information questionnaire about their level of physical activity, sport activity and upper limb dominance (defined as the arm preferred for throwing) (Borms & Cools, 2018). All anthropometric measurements were made by a single researcher: (i) body mass and height were obtained using a stadiometer (model 220; Seca, Germany), and (ii) the upper limb length was measured while the shoulder was abducted to 90°, the elbow extended and the wrist and hand in neutral. Measurements from the C7 spinous process to the tip of the longest digit were then taken. The study design considered the evaluation of the shoulder AJPS and three upper extremity functional performance tests: YBT-UQ, CKCUEST and SMBT. The above tests were performed by a single evaluator, with 10 years of experience as a physical therapist and who participated in a 24-h familiarization session for the above procedures. This training was based on the standardization of the protocols and the identification of execution errors. Prior to the evaluation of the upper extremity functional performance tests, a 5-min warm-up was carried out, consisting of static stretching exercises (two stretches per muscle) for the shoulder complex muscles (upper trapezius, shoulder rotator muscles, biceps brachii, triceps brachii, and pectoralis major), as this type of stretching has not been shown to influence the repositioning accuracy of joint position sense (Moradi et al., 2020; Torres, Duarte & Cabri, 2012).

***Active Joint Position Sense*.** Shoulder proprioception was quantified using an AJPS protocol (passive/active, *i.e*., passive demonstration with active reproduction) with an inclinometer (Baseline Digital Inclinometer: Fabrication Enterprises, Elmsford, NY, USA) in three positions: (i) 90° of shoulder flexion (90°Flex), (ii) 90° of shoulder internal rotation at 90° of abduction (90°IR/ABD), and (iii) 90° of shoulder external rotation at 90° of abduction (90°ER/ABD) (Dover & Powers, 2003; Gumina et al., 2019; Vafadar, Cote & Archambault, 2016). A recent systematic review (Ager et al., 2017) reported that the passive/active protocol had a strong intra-session reliability (weighted average ICC = 0.92 ± 0.1, *n* = 204), as well as good reliability when using a digital inclinometer (weighted average ICC = 0.84, *n* = 56). Furthermore, this protocol has been shown to be valid (criterion validity) when compared to the Vicon motion capture system (*r* = 0.80) (Dover & Powers, 2003; Vafadar, Cote & Archambault, 2016).

The evaluation was performed with the participants in a seated position and blindfolded, without noise cancelling headphones. Male participants were bare chested and female participants wore a tank top, and those with long hair were advised to tie their hair above shoulder height. The movements of the dominant arm were evaluated from the starting position to the target position. For the AJPS at 90°Flex, the initial position was the anatomical position (0°); and for the AJPS at 90°IR/ABD and 90°ER/ABD, the initial position was 90° of shoulder abduction and 90° of elbow flexion. Firstly, an evaluator placed one of its hands on the distal aspect of the humerus (above the lateral epicondyle) and the other on the distal aspect of the radius and ulna of the participant. After which, they passively moved the participant’s arm to the predetermined target position, held this position for 5 s (displayed by a timer), and subsequently passively returned the limb to the starting position. After 5 s, the blindfolded participant was asked to actively reproduce the shown target position. Participants acknowledged verbally when they felt they had achieved the target angle. Prior to testing, each participant performed a single AJPS familiarization practice movement to 60° of shoulder abduction. The outcome variable was the absolute angular error (i.e., proprioception error), which was the absolute difference between the target angle and the participant’s reproduced angle in degrees. Three attempts for each joint angle were completed and the average proprioception error was taken for each target angle. Ten seconds of rest was given between testing trials. To avoid any learning effect, no corrections were provided during testing and the order of target angles was randomly allocated with a randomization software. To determine the intra-rater reliability of the protocol, the three measurements of each target angle were used for psychometric testing.

***Y-Balance Test-Upper Quarter (YBT-UQ)*.** This upper-extremity functional performance test is performed in a closed-kinetic chain position that allows the assessment of strength, stability, and mobility (Cook, 2010; Gorman et al., 2012). This test supports good to excellent test-retest reliability in different reaching directions (superolateral direction, ICC = 0.91–0.99; medial direction, ICC = 0.82–0.98; inferolateral direction, ICC = 0.80–0.98) (Gorman et al., 2012; Schwiertz et al., 2019). The YBT-UQ uses a YBT Kit (Move2Perform, Evansville, IN, USA), which consists of a stance platform, to which three pipes are attached in the superolateral, medial and inferolateral directions. There is a 90° angle between the superolateral and inferolateral directions, and 135° between each of these with the medial direction, forming a Y-shape. The dominant hand is placed on the stance platform with the thumb placed behind the red line. The participant adopts a three-point plank position with the dominant upper extremity perpendicular to the hand, with their feet shoulder-width apart. With the unsupported free hand, the participant pushes a reach indicator as far as possible in the medial, inferolateral and superolateral directions, and then returns to the starting position under controlled movements (Borms, Maenhout & Cools, 2016; Borms & Cools, 2018; Gorman et al., 2012). The trial is to be repeated if the participant fails to maintain three-point contact, pushes the indicator out of reach or if the ground or reach indicator is used for additional support (Borms & Cools, 2018). After two familiarization practices, three trials were performed for each direction, with a 30-s rest between each trial. To avoid any learning effect, the order of reach directions was randomly allocated with a randomization software. For each reach direction, the average distance (i.e., absolute value in centimetres (cm)) was calculated and normalized for upper limb length (a relative percentage). Lastly, the YBT-UQ composite score –an indicator of the overall test performance– was calculated, averaging the distances of the three normalized directions. The three measurements of each YBT-UQ distance as well as the composite score were used to determine intra-rater reliability of this tool.

***Closed Kinetic Chain Upper-Extremity Stability Test (CKCUEST)*.** This upper-extremity functional performance test is also performed in a closed-kinetic chain position and has been shown to be a reliable (test re-test reliability ICC = 0.82–0.98) method to assess power, velocity, and stability (Borms & Cools, 2018; Declève, Van Cant & Cools, 2021; Goldbeck & Davies, 2000; Tucci et al., 2014). The participant adopts a four-point position on the floor, with their hands 91.4 cm apart (marked with two parallel strips of adhesive tape on the 30 cm floor) and with their shoulder’s perpendicular to their hands. Previous studies have shown that there are no kinetic and kinematic differences in the performance of this test, if the separation distances between the hands are different (*i.e*.: 91.4 cm (original), inter-acromial, and 150% inter-acromial distances) (Tucci et al., 2017). As a starting position, the spine and lower limbs of the participant are aligned, and their feet are separated shoulder-width apart. During the 15-s of testing, participants are to move one hand to touch the dorsum of the opposite hand, and then returned the moving hand back to the starting position. Subsequently, the participant performed the same movement with the other hand. Participants were instructed to perform as many alternating touches as possible, while maintaining the correct four-point position (Borms & Cools, 2018). If one of the touches did not reach the required distance of the test, the testing continued; however, the trial was not counted (Guirelli et al., 2021; Tucci et al., 2014). After one familiarization trial, three maximal-effort trials were executed with a 45-s rest between sets. The average number of touches of the three maximal-effort trials were calculated and were normalized by height in meters (touches/m) to each participant (Declève, Van Cant & Cools, 2021). The three normalized trials were used to determine intra-rater reliability of this tool.

***Seated Medicine Ball Throw (SMBT)*.** This upper extremity functional performance test is performed in an opened-kinetic chain movement and has been shown to be a reliable (test re-test reliability ICC = 0.88–0.99) method to assess unilateral and bilateral upper body power and strength (Borms & Cools, 2018; Harris et al., 2011; van den Tillaar & Marques, 2013). A 10-meter (m) tape measure was placed on the floor and the end was fixed to the wall. The medicine ball was covered in gymnastic chalk to leave a clear mark on the floor, allowing an easy measurement of the throwing distance for each trial. Participants sat on the floor with their legs out-stretched, and their head, shoulders and back against the wall. For the bilateral SMBT, the participants held a 2-kilogram (kg) medicine ball with shoulders in 90° abduction and elbows flexed, so that the ball was placed at the centre of their chest. The participant then pushed the ball away from the centre of their chest in a straight line as far as possible, using a motion similar to a basketball chest pass (Borms & Cools, 2018; Cigercioglu et al., 2021; Declève, Van Cant & Cools, 2021; Harris et al., 2011). For the unilateral component of the test, participants held a 2 kg medicine ball with their dominant hands and placed their shoulders in 90° abduction (in the scapular plane) and their elbows in 90° flexion. Then, the participants were asked to throw the medicine ball horizontally and far as possible (Cigercioglu et al., 2021). The correct throwing technique was monitored by an evaluator. If it was not correct, the participant had to repeat the trial. After two familiarization trials, three maximal-effort trials were executed with a 1-min rest between each set, in order to avoid a fatigue effect. To account for the difference in arm lengths, the medicine ball was dropped by the participant, with their arms extended in front of the body. The distance between the wall and the most proximal end of the chalk mark from this drop, was subtracted from the total throwing distance (Borms & Cools, 2018). The average throwing distance of the three maximal-effort attempts (cm) was calculated. The three trials were used to determine intra-rater reliability of this tool.

### Statistical analysis

Descriptive statistics (means ± standard deviations, spread) were calculated across participants for each outcome measure: AJPS, YBT-UQ, CKCUEST, and SMBT, respectively. Standard error of the measurement (SEM) and intraclass correlation coefficient (ICC; two-way random, absolute agreement, average measure) were calculated for the intra-rater reliability of each outcome measure. The normality of distribution was evaluated using the Kolmogorov-Smirnov test. The following statistical analysis were carried out: (i) the relationship between AJPS and YBT-UQ, CKCUEST, and SMBT was analyzed using the Pearson’s product correlation coefficient (r) was used to evaluate the relationship between variables. A correlation coefficient (r) from 0 to 0.4 was considered “weak”, 0.41 to 0.7 as “moderate” and 0.71 to 1.0 as a “strong” association (Cohen, 1988); and (ii) a stepwise multiple linear regression analysis was used to identify if there are any associations between AJPS and the YBT-UQ, CKCUEST or SMBT performances. The models were adjusted for sex, age, body mass, height, and upper extremity length. For this method, the outcomes that demonstrated a simple significant correlation (from highest to lowest correlation), were consecutively added to this model. This selection step was introduced to limit the number of factors. The collinearity of the data was verified through the identification of the variance inflation factor (VIF), opting to eliminate the variables that showed collinearity with a VIF > 10, in order to define the definitive multiple linear regression model. The coefficient of determination (R^2^) was used to interpret the meaningfulness of the relationships. IBM SPSS® Software (version 25.0 for Mac; Armonk, NY, USA) was used to perform the statistical analyses of the data. In all tests, an alpha level of 0.05 was applied. GraphPad Prism version 8.0.0 software (GraphPad Software, San Diego, CA, USA) was used to design the plots of predicted values against the standardized residuals (Z-score) obtained from the multiple linear regression models.

## Results

Fifty-two participants were included in the analysis and all data presented with a normal distribution. Table 2 summarizes the shoulder proprioception and functional test outcomes and the associated intra-rater reliability. The results demonstrated good intra-rater reliability (ICC = 0.79–0.89) for AJPS measures and excellent intra-rater reliability (ICC = 0.92–0.98) for all the upper-extremity functional performance tests.

**Table 2:** Shoulder proprioception and functional test outcomes with their associated intra-rater reliability.

	Total (*n* = 52)				
	Mean ± SD	ICC	95% ICC		SEM
AJPS at 90°Flex (°)	3.6 ± 2.2	0.79	0.68	0.87	0.3
AJPS at 90°IR/ABD (°)	7.1 ± 4.6	0.89	0.82	0.93	0.6
AJPS at 90°ER/ABD (°)	4.9 ± 3.2	0.88	0.81	0.92	0.4
YBT-UQ Superolateral (%)	65.5 ± 10.1	0.96	0.94	0.98	1.4
YBT-UQ Medial (%)	105.2 ± 9.5	0.94	0.91	0.96	1.3
YBT-UQ Inferolateral (%)	80.8 ± 11.8	0.96	0.94	0.98	1.6
YBT-UQ Composite score (%)	83.8 ± 8.4	0.95	0.92	0.96	1.2
CKCUEST (touches/m)	8.9 ± 1.6	0.92	0.88	0.95	0.2
SMBT Dominant limb (cm)	245.0 ± 68.9	0.95	0.91	0.98	9.6
SMBT Bilateral (cm)	309.8 ± 73.9	0.98	0.96	0.99	13.0

**Note:**

AJPS, Active Joint Position Sense; 90°Flex, 90° of flexion; 90°IR/ABD, 90° of internal rotation at 90° of abduction; 90°ER/ABD, 90° of external rotation at 90° of abduction; YBT-UQ, Y-Balance Test Upper Quarter; CKCUEST, Closed Kinetic Chain Upper Extremity Stability Test; SMBT, Seated Medicine Ball Throw; ICC_2_,_k,_ Intraclass Correlation Coefficients with 95 % confidence interval; SEM, standard error of measurement. SEM is provided in raw units (AJPS and SMBT) and percentages (YBT-UQ and CKCUEST).

AJPS measurements at 90° of internal rotation showed the greatest number of significant correlations with the upper-extremity functional performance tests (see Table 3); of which the inferolateral YBT-UQ (*r* = −0.57) and the YBT-UQ composite score (*r* = −0.43) demonstrated the highest magnitude (moderate) correlations. Furthermore, AJPS at 90°ER/ABD demonstrated a weak significant correlation with the SMBT test (*r* = −0.30). Lastly, AJPS at 90°Flex did not show any correlations with any of the evaluated sport performance tests for the upper extremities.

**Table 3:** Correlations between active joint position sense and upper extremity functional performance tests.

	AJPS 90°Flex		AJPS 90°IR/ABD		AJPS 90°ER/ABD	
	*r*	*p*-value	*r*	*p*-value	*r*	*p*-value
YBT-UQ Superolateral	−0.02	0.878	−0.31	0.025	−0.10	0.476
YBT-UQ Medial	−0.04	0.755	−0.11	0.458	−0.09	0.513
YBT-UQ Inferolateral	−0.11	0.434	−0.57	0.001	−0.07	0.645
YBT-UQ Composite score	−0.08	0.588	−0.43	0.002	−0.11	0.456
CKCUEST	0.19	0.174	−0.01	0.992	−0.07	0.639
SMBT Dominant limb	−0.05	0.703	−0.25	0.071	−0.30	0.032
SMBT Bilateral	−0.09	0.528	−0.30	0.031	−0.23	0.099

**Note:**

AJPS, Active Joint Position Sense; 90°Flex, 90° of flexion; 90°IR/ABD, 90° of internal rotation at 90° of abduction; 90°ER/ABD, 90° of external rotation at 90° of abduction; YBT-UQ, Y-Balance Test Upper Quarter; CKCUEST, Closed Kinetic Chain Upper Extremity Stability Test; SMBT, Seated Medicine Ball Throw.

**Figure 1:**
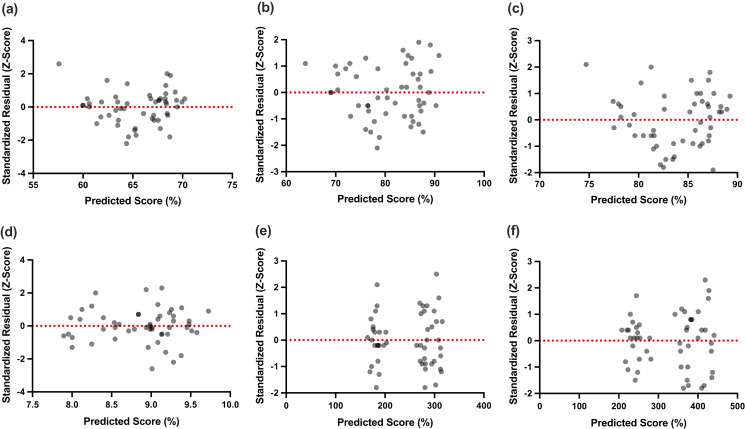
Predicted values against the standardized residuals (Z-score) of sport performance tests scores obtained from the multiple linear regression analysis. (A) YBT-UQ Superolateral, (B) YBT-UQ inferolateral, (C) YBT-UQ Composite score, (D) CKCUEST, (E) SMBT Dominant limb, (F) SMBT bilateral. MLR, multiple linear regression; YBT-UQ, Y-Balance Test Upper Quarter; CKCUEST, Closed Kinetic Chain Upper Extremity Stability Test; SMBT, Seated Medicine Ball Throw.

Stepwise multiple linear regression analysis revealed that AJPS at 90°IR/ABD was associated with the superolateral, inferolateral, and composite score of YBT-UQ (Table 4). Of these associations, the inferolateral YBT-UQ was the upper-extremity functional performance test that presented with the highest percentage of change (%) in R^2^ due to the AJPS at 90°IR/ABD (%R^2^ = 0.32; β = −1.5; *p* = 0.001). Moreover, sex and body mass were associated with dominant limb and bilateral SMBT. Figure 1 outlines the relation between the predicted and standardized residuals (i.e., Z-score) of sport performance tests scores obtained from the multiple linear regression analysis. No outcomes were found to be associated with the medial YBT-UQ or CKCUEST performances.

**Table 4:** Stepwise multiple linear regression model of upper extremity functional performance tests adjusted for: active joint position sense, age, body mass, height, and upper limb length.

		R²	%R^2^*	β	SE	95% CI		*p*-value
YBT-UQ Superolateral	Constant			70.3	2.5	65.4	75.3	
	AJPS at 90°IR/ABD	0.10	0.10	−0.7	0.3	−1.3	0.1	0.025
YBT-UQ Inferolateral	Constant			91.1	2.5	86.1	96.2	
	AJPS at 90°IR/ABD	0.32	0.32	−1.5	0.3	−2.1	−0.8	0.001
YBT-UQ Composite score	Constant			89.4	2.0	85.4	93.4	
	AJPS at 90°IR/ABD	0.18	0.18	−0.8	0.2	−1.3	−0.3	0.002
CKCUEST	Constant			12.3	1.6	9.1	15.6	
	Body mass	0.08	0.08	−0.05	0.02	−0.10	−0.01	0.037
SMBT Dominant limb	Constant			79.6	45.2	−11.3	170.4	
	Sex	0.61	0.61	91.3	13.7	63.7	118.9	0.001
	Body mass	0.65	0.04	1.6	0.7	0.2	3.0	0.024
SMBT Bilateral	Constant			45.3	54.0	−63.1	153.8	
	Sex	0.65	0.65	119.2	16.4	86.2	152.1	0.001
	Body mass	0.73	0.08	3.1	0.8	1.4	4.7	0.001

**Notes:**

SE, standard error; CI, confidence interval; AJPS, Active Joint Position Sense; 90°Flex, 90° of flexion; 90°IR/ABD, 90° of internal rotation at 90° of abduction; 90°ER/ABD, 90° of external rotation at 90° of abduction; YBT-UQ, Y-Balance Test Upper Quarter; CKCUEST, Closed Kinetic Chain Upper Extremity Stability Test; SMBT, Seated Medicine Ball Throw.

* %R^2^ represents the percentage of variability (change) that explains the consecutive addition of each of the independent variables to the regression model.

## Discussion

Although there have been previous studies that evaluated the association between some motor control variables, such as the strength of the shoulder complex musculature and the performance of upper-extremity functional tests (Guirelli et al., 2021; Mendez-Rebolledo et al., 2022; Westrick et al., 2012); to our understanding, there has yet to be research conducted on the association between proprioceptive acuity and the functional performance of sports tests in both closed and opened-kinetic chain tasks. Our results suggest that the AJPS of 90°IR/ABD and 90°ER/ABD were the only proprioceptive variables related to the YBT-UQ and SMBT, while no relationship was observed with the CKCUEST test (see Table 2). Despite these correlations, the AJPS at 90°IR/ABD was the only proprioceptive variable that held an association with the performance of the YBT-UQ (superolateral, inferolateral and composite scores).

Our results show that an intact shoulder repositioning capability is relevant to upper extremity functional performance, demonstrated by the ability of AJPS at 90°IR/ABD to explain a variance between 10 and 32% of YBT-UQ performance (see Table 4). These results can be explained by the potentially higher firing (activation) rates of the proprioceptors in a weight-bearing position, due to a smaller base of support in a three-support position compared to a four-support (Cools et al., 2015; Dhahbi et al., 2018; Gorman et al., 2012; Pontillo et al., 2007). During the inferolateral reach of the YBT-UQ test, the upper extremity in an open-kinetic chain crosses the body between the chest and the contralateral upper extremity; the upper limb in weight-bearing position (with the shoulder in internal rotation and adduction) displaces the center of mass towards itself (Gorman et al., 2012; Westrick et al., 2012), generating a greater requirement for proprioceptive information in internal rotation of the glenohumeral joint. In this context, it has been suggested that the compression of a joint and surrounding soft tissue during a weight-bearing or loaded position, can cause mechanical deformation of localized mechanoreceptors, including proprioceptors, thus causing a heightened sense of proprioception (Bang et al., 2015; Lee et al., 2014). Therefore, it is understandable that we see a strong and positive correlation between AJPS in internal rotation and the upper extremity performance tests of the YBT-UQ and SMBT, as all three outcome measures could be detecting a heightened sense of shoulder proprioception amongst volleyball players. Our results suggest a possible association between the sense of proprioception and upper extremity motor control, in both open and closed-chained tasks, which is in line with the current literature concerning both healthy (Guirelli et al., 2021) and pathological shoulders (Balke et al., 2011; Clark, Röijezon & Treleaven, 2015; Lubiatowski et al., 2019).

The previous explanation assumes that volleyball players have a musculoskeletal system without sport-specific adaptations. In this sense, our results can also be partially explained by the known GH internal rotation deficit and tightness experienced by volleyball players (Harput et al., 2016; Telles et al., 2021). Due to the repetitive overhead rotational motion during spiking, serving, and blocking, it is well documented that volleyball players often develop excessive external rotation range, and limited internal rotation range. With repetitive movements and micro-trauma, connective tissues can experience deformation, structural changes and may have an altered feedback mechanism for peripheral senses, such as proprioception (Chen et al., 2021; Langevin, 2021). A recent systematic review on the effects of rehabilitation of shoulder proprioception suggests a specificity to proprioception deficits (Ager et al., 2021); meaning specific pathologies (shoulder instabilities or rotator cuff dysfunctions, for example) present with specific proprioception deficits. This same argument could be made for the performance side of proprioception. Specialized athletic training, in internal rotation in the case of volleyball players, could lead to a specific, or improved sense of shoulder proprioception, found with specific movements or activities. Therefore, it is understandable that we see a heightened sense of AJPS in internal rotation with volleyball players, which demonstrated a strong correlation to functional testing in a weight-bearing (YBT-UQ) or a loaded condition (SMBT).

Nonetheless, shoulder proprioception should not be the only factor to which an important role is attributed during the performance of the YBT-UQ, but consideration should also be given to other physical and motor capacities. Previous research has observed significant relationships between the isometric strength of the rotator cuff, scapular, and stability muscles with the different reaching directions of the YBT-UQ (Borms, Maenhout & Cools, 2016; Guirelli et al., 2021; Westrick et al., 2012), suggesting that the performance of this functional test depends on the ability of the anterior muscles to control and distribute body mass along the spine, shoulder, and elbow segments to minimize the load placed on the distal joints (Guirelli et al., 2021). For all of aforementioned reasons, future research should explore and consider the possible role of these factors in the performance of the YBT-UQ, especially the isometric strength (e.g., CORE muscles, scapular muscles, and shoulder rotator muscles) and joint position sense during the performance of the YBT-UQ test.

The present study suggests that body mass and sex are factors significantly associated with SMBT performance, explaining a variance between 65 and 73% of the model. It has been observed that muscle mass and strength are influenced by gonadal steroid hormones, specifically by a higher circulating testosterone in male, exceeding 15-fold that of female at any age, which could explain the greater development of strength and power during the execution of an SMBT (Handelsman, Hirschberg & Bermon, 2018). On the other hand, any association between AJPS at 90° external rotation and the SMBT of the dominant limb was not observed, despite some researchers indicating that the functional performance of the shoulder is susceptible to the ability to reposition the glenohumeral joint in a 90° external rotation angle, seeing as this position is considered fundamental for the force production and transfer in the cocking phase of the throwing and attacking in several sports, including volleyball (DiCesare et al., 2019; Sarvestan, Svoboda & Linduška, 2020).

Lastly, our research did not reveal a relationship between the AJPS and the CKCUEST. Possibly, the CKCUEST test does not require, to a greater extent, AJPS in rotation or shoulder flexion, since the movements required in this test are mainly horizontal shoulder adduction (Borms & Cools, 2018; Taylor et al., 2016; Tucci et al., 2014). This finding also strengthens the argument of a possibility of seeing specific proprioception gains, in line with the specific demands and training of the sport; theorized to be in internal rotation for volleyball players. It is also possible that this upper extremity functional performance test is representative of other motor capabilities such as strength and power (Borms & Cools, 2018; Taylor et al., 2016; Tucci et al., 2017); as a moderate to strong significant relationship between the isometric strength of the shoulder abductors, lower trapezius and trunk flexors has recently been reported with the CKUEST (Guirelli et al., 2021).

***Strength and Limitations*.** This cross-sectional study has investigated two important aspects of sport performance for volleyball players, notably shoulder proprioception and functional performance. Strengths of this study include a good sample size and the psychometric reporting of AJPS protocols and functional tests for the upper extremities. Our results suggest good to excellent intra-rater reliability (ICC = 079 to 0.98), with small errors of measurement for the evaluations of AJPS (SEM = 0.3 to 0.6°) YBT-UQ (SEM = 1.2 to 1.6 %), CKCUEST (SEM = 0.2 touches/m), and SMBT (SEM = 9.6 to 13.0 cm), respectively. The reliability results internally validated the data registry and subsequent correlation and multiple linear regression analyses. Furthermore, our findings are similar to those observed by Borms & Cools (2018), who provided reference values for volleyball players in a similar age range to this study (Borms & Cools, 2018); with the exception of the CKCUEST scores, which in the present investigation, was normalized by the height of the participants following the recommendations of previous research (Decleve et al., 2020).

However, for the advancement of knowledge in this area, a few limitations should be addressed for future studies. Our study employed a passive/active protocol for the evaluation of AJPS, which may not support the strongest ecological validity (Gibson, 1979) nor be the best representation of upper-extremity functional performance for volleyball players. The use of an active demonstration/active production (active/active) protocol could have been more suitable for this population. Although a recent systematic review on shoulder proprioception suggests that an active/active protocol has the weakest intra-session reliability (weighted average ICCs = 0.34) of all AJPS protocols (Ager et al., 2017). It is clear that the measurement of shoulder proprioception continues to be a complex sensorimotor sense to quantify. For these reasons, it is necessary to deepen our knowledge of the various proprioceptive sub-modalities and their possible role in motor control and sport performance in both open and close-kinetic chain activities.

For future studies we recommend the use of normalized scores for the various sports performance tests, based on anthropometric measures that directly impact performance, such as upper limb length for the YBT-UQ and SMBT scores; and height and body mass for the CKCUEST and SMBT scores (Borms & Cools, 2018; Decleve et al., 2020; Gorman et al., 2012).

## Conclusions

AJPS evaluated at 90° of internal rotation was the only proprioception variable with an association to an upper extremity functional test, more specifically, the YBT-UQ test. Further research is required to consider how other proprioception sub-modalities may influence the performance of the upper extremities during functional testing. Our findings suggest a possible expanded role for proprioception, as a contributing feedback and feed-forward mechanism in upper limb motor control. Nevertheless, the relationship between shoulder proprioception and upper extremity functional performance tests continues to be an area worth further exploration. Our results are encouraging and reinforces the message to clinicians that shoulder proprioception needs to be a part of their rehabilitation strategy and considered to be a prognostic milestone for volleyball players to return to sport.

## Supplemental Information

10.7717/peerj.13564/supp-1 Supplemental Information 1 Raw Data.

## Additional Information and Declarations

### Data Availability

The following information was supplied regarding data availability: The raw measurements are available in the Supplemental File.
